# Factors Associated with Long-Term Dietary Supplement Use among Korean Breast Cancer Survivors: A Cross-Sectional Study

**DOI:** 10.3390/nu15184087

**Published:** 2023-09-21

**Authors:** Seonghye Kim, Yohwan Yeo, Jinyoung Shin, Dong Wook Shin, Belong Cho, Yun-Mi Song

**Affiliations:** 1 Department of Family Medicine, Samsung Medical Center, Sungkyunkwan University School of Medicine, Seoul 06351, Republic of Korea; shye.kim7@gmail.com (S.K.); dongwook.shin@samsung.com (D.W.S.); 2 Department of Family Medicine, Hallym University Dongtan Sacred Heart Hospital, Hwaseong 18450, Republic of Korea; hanyoung29@naver.com; 3 Department of Family Medicine, Research Institute on Healthy Aging, Konkuk University Medical Center, Konkuk University School of Medicine, Seoul 05030, Republic of Korea; doctorsjy@naver.com; 4 Department of Family Medicine & Health Promotion Center, Seoul National University Hospital, Seoul National University College of Medicine, Seoul 03080, Republic of Korea; belong@snu.ac.kr

**Keywords:** breast neoplasms, cancer survivors, dietary supplements, health behavior

## Abstract

Purpose: The factors associated with the dietary supplement (DS) use of Asian breast cancer survivors in consideration of the duration of use and types of DS have not been well established. Methods: We recruited 693 Korean female breast cancer survivors at two university-affiliated hospitals and collected study data through a self-administered questionnaire and a review of medical records. A multiple logistic regression analysis was conducted to evaluate the multivariable-adjusted association between DS use and study variables. Results: The prevalence of any (≥2 weeks) and long-term (≥6 months) DS use among study participants was 48.2% and 12.0%, respectively. Education level, alcohol use, adequate physical activity (≥150 min/week), and time lapse after cancer diagnosis were positively associated with any DS use. Among DS users, as compared with short-term (≥2 weeks and <6 months) users, long-term users were more likely to have a higher cancer stage, more diverse cancer treatment modalities, a shorter time since cancer diagnosis, and lower fear of cancer recurrence. When we repeated the analysis for each DS type, time lapse after cancer diagnosis showed a consistently inverse association with long-term use of the most frequently consumed DS (multivitamins, followed by vitamin D/calcium, vitamin C, and omega-3). The number of cancer treatment modalities was positively associated with the long-term use of multivitamins and vitamin D/calcium. Alcohol consumption and low bone mineral density were positively associated with long-term vitamin D/calcium use. Conclusions: The factors associated with DS use differed by the duration of DS use and specific DS type. Long-term DS use was more frequently associated with cancer-related factors.

## 1. Introduction

Breast cancer is the most frequently reported malignancy among women worldwide. According to the Global Cancer Observatory of the International Agency for Research on Cancer (IARC), 11.7% of all cancer patients were diagnosed with breast cancer in 2020 [1]. In Korea, breast cancer accounted for approximately 21.1% of all female cancer cases, with an estimated 24,806 new cases in 2020 [2]. The advancement of early detection modalities and the improvement of treatment options have contributed to an increase in survival rates of breast cancer patients, which, in turn, has resulted in a rapidly increasing number of breast cancer survivors. In Korea, the number of female breast cancer survivors was estimated to be 278,953 in 2020 [2].

Cancer survivors frequently suffer from a wide range of chronic health problems affecting their physical, psychological, and social functions, which are related to primary cancers as well as the late effects of cancer treatments [3,4]. Therefore, the transition from active treatment to long-term post-treatment care is necessary for cancer survivors [4]. In the course of the transition, cancer survivors struggle to overcome various barriers and try to satisfy unmet needs for resumption of their normal activities, employment, and social roles [5]. Although a large part of cancer survivorship care is provided by medical professionals, many survivors actively consider modifying their health behaviors [6,7,8].

Dietary supplement (DS) use is one of the common health behaviors of cancer survivors, along with consumption of healthy foods, physical exercise, smoking cessation, and abstaining from drinking. Studies in Western countries have reported that more than half of breast cancer survivors were DS users [8,9,10,11]. Sociodemographic and disease-related characteristics such as female sex, higher education status, higher income, not smoking, not being obese, engaging in moderate physical activity, and believing in the function of DS for the prevention of cancer recurrence have been found to be associated with the DS use of cancer survivors in Western countries [9,11,12,13].

However, studies to evaluate the prevalence and factors associated with the long-term DS use of Asian breast cancer survivors have been scarcely conducted, and have had controversial findings. In a Korean population-based study, the prevalence of DS use among breast cancer survivors was 55.9%, which was higher than the rate of DS use among all cancer survivors (33.3%) and cancer-free individuals (22.1%) [14]. On the other hand, in our previous study of 1852 Korean survivors of diverse cancers, the rate of long-term DS use (≥6 months) was 15.7%. We believe that the inconsistency in the DS use rate between the two Korean studies is related to the different operational definitions of DS use by duration (any vs. 6 months or longer), given that adherence to medication tends to decrease over time.

Regarding the factors associated with DS use, a population-based Korean study showed that higher education level, moderate physical activity, higher circulatory vitamin D level, and low vegetable consumption were associated with DS use in female cancer survivors [14]. A separate analysis of breast cancer survivors was not performed in this Korean study, probably because of the small number of breast cancer survivors (59 women) included in the study [14]. In our previous study, being female was related to a higher probability of long-term DS use, and among female cancer survivors, breast cancer survivors were less likely to take DS compared with female survivors of other cancers such as stomach cancer [15]. This finding pointed to the necessity of further examining the patterns and related factors of DS use by breast cancer survivors.

Therefore, we conducted this study to investigate the DS use of breast cancer survivors in more detail. We evaluated the rate of DS use and the factors associated with DS use according to the duration of DS use and specific type of DS.

## 2. Methods

### 2.1. Study Participants

The study participants were female Korean breast cancer survivors who were recruited from the cancer survivorship clinics of two university-affiliated hospitals in the Republic of Korea between September 2014 and February 2017. The breast cancer survivors visited the clinics with various health issues, including surveillance of cancer recurrence and secondary cancer screening. Among the initially recruited 792 breast cancer survivors, we excluded 99 women for the following reasons: lack of data on DS use (2 women), absence of data on surgery (6 women), missing information on cancer stage or time at diagnosis (19 women), cancer recurrence (18 women), and diagnosis of secondary cancer (54 women). We excluded survivors whose surgical treatment history was not verified, because surgery is regarded as the main treatment modality for breast cancer, and breast cancer patients who receive only chemotherapy or CCRT are very rare, unless there is a metastasis [16,17]. Ultimately, a total of 693 female breast cancer survivors were included in the analysis. The study protocol was approved by the Institutional Review Board (IRB) of each hospital, and informed written consent was obtained from all study participants. All research was performed in accordance with the study protocol.

### 2.2. Study Variables

We collected information on DS use after cancer diagnosis using a self-administered questionnaire. To estimate DS usage status, we asked the study participants the following questions: (1) Have you ever taken DS regularly, at least once a week over 2 weeks or longer after your cancer diagnosis? and (2) If your answer was ‘yes’ to the first question, please provide details on the type, frequency and duration of your DS use. If a participant had taken more than one DS, they were requested to provide detailed information on each type of DS. To obtain information on the types of DS, we provided a list of DS known to be commonly consumed among the Korean population such as multivitamins, vitamin C, vitamin D, calcium, iron, omega-3, and coenzyme Q10. Study participants could select all the DS they had used from the list and could write down the specific name of a DS if it was not included in the list. Iron supplementation for treating iron deficiency anemia was not counted as DS use. In this study, we defined any DS use as consuming DS regularly, at least once a week for 2 weeks or longer. Then, we defined short-term DS use as taking DS for between 2 weeks~<6 months, and long-term DS use as taking DS for 6 months or longer, respectively.

Data on cancer-related clinical characteristics were collected by reviewing medical records, which included cancer stage, time at breast cancer diagnosis, cancer treatment modality, and bone mineral density (BMD). We calculated the time interval between survey time and time at cancer diagnosis, and we categorized the time since breast cancer diagnosis into four groups: ≤1 year, 1–5 years, 6–10 years, and >10 years. We counted the total number of combined cancer treatment modalities: surgical treatment and/or other treatment (chemotherapy, radiotherapy, and hormonal therapy). BMD, measured using dual-energy X-ray absorptiometry, was categorized into two groups based on the lowest T-score of BMD at the lumbar spine or left femur: normal (T-score ≥ −1.0) or low BMD (T-score < −1.0).

Data on sociodemographic and health behavioral characteristics were collected using a self-administered questionnaire. We categorized the monthly household income level into three groups: <KRW 2,000,000, KRW 2,000,000–3,999,999, and ≥ KRW 4,000,000. The education level was grouped into three levels: ≤middle school graduate, high school graduate, and ≥college graduate. We categorized marital status into two groups: living with a spouse/partner or not. Menopausal status was categorized into two groups: postmenopausal and premenopausal. Among the participants who reported no menstrual cycle within the past 12 months, only those who were aged 55 years or older, experienced natural menopause, received estrogen replacement therapy, or underwent iatrogenic menopause due to chemotherapy, hormonal treatment, or bilateral oophorectomy were regarded as having postmenopausal status. Family history of cancer was also categorized into two groups: yes or no. We checked the presence of comorbid disease by asking if participants had been diagnosed with any of the following diseases: cerebrovascular disease, cardiovascular disease, chronic respiratory disease, chronic liver disease, chronic renal disease, hypertension, diabetes, dyslipidemia, anemia, thyroid disease, osteoporosis, lymphedema, peripheral neuropathy, and psychiatric disorder. Previous receipt of lifestyle modification counseling with health care providers was divided into two groups: yes or no. We categorized smoking status into never-smokers and ever-smokers. Alcohol consumption was categorized into current non-drinkers or drinkers. We defined adequate physical activity as engaging in moderate-intensity activity regularly, more than 150 min per week, according to the physical activity guidelines of the American Cancer Society for cancer survivors [18].

We assessed the psychological status and quality of life of the study participants including anxiety, depression, and fear of cancer recurrence by applying the Hospital Anxiety and Depression Scale (HADS), Fear of Cancer Recurrence Inventory-Short Form (FCRI-SF), and EuroQoL Visual Analogue Scale (EQ-VAS). HADS is a self-assessment tool used to evaluate anxiety and depression states in an outpatient clinic setting. The states of anxiety and depression were defined by applying cut-off values of 8 or higher for the score of HADS-A/HADS-D [19,20]. The FCRI-SF severity subscale reflects the presence and severity of FCR. We applied an empirically validated cut-off value of 13 or higher for screening a clinically significant level of FCR [21]. EQ-VAS is a scale for measuring current health-related quality of life, which ranges from 0 (worst imaginable) to 100 (best imaginable) [22]. In this research, the EQ-VAS level was classified into two groups: ≤70 or >70, considering the median value was 70 and the mean value was 67.3 among study participants.

### 2.3. Statistical Analysis

We presented descriptive statistics for the prevalence of any and long-term DS use for the nine most commonly used DS. We compared the sociodemographic, cancer-related, health behavioral, and psychological characteristics between users of any DS and non-users, as well as between short-term DS users and long-term DS users. We used chi-square tests or Fisher’s exact tests for categorical variables after excluding participants with missing values for the relevant variables. In addition, we conducted the Mantel–Haenzel chi-square test to evaluate the presence of statistically significant trends for ordinary variables. We estimated odds ratios (OR) and 95% confidence intervals (95% CI) for each variable associated with any use and long-term use of DS via multiple logistic regression analyses. We included age, education level, menopausal status, BMD, family history of cancer, cancer stage, time since cancer diagnosis, number of cancer treatment modalities, comorbid diseases, anxiety, depression, FCR, EQ-VAS, lifestyle modification counseling, alcohol consumption, and physical activity as covariates in the regression model. We selected covariates considering the statistical significance level (*p* < 0.1) estimated from the univariate analysis of our study or by referring to other studies. We further estimated OR (95% CI) for each variable associated with any use and long-term use of the four most commonly consumed DS by multiple logistic regression analyses. All statistical analyses were conducted by SAS ver. 9.4 (SAS Institute Inc., Cary, NC, USA). Two-sided tests were performed, with the level of statistical significance set at <0.05.

## 3. Results

Table 1 shows the distribution of DS use among study participants. Among 693 Korean adult breast cancer survivors, the prevalence of any and long-term DS use was 48.2% and 12.0%, respectively. The most frequently consumed DS was multivitamins, followed by vitamin D/calcium, vitamin C, and omega-3.

Table 2 shows the findings from comparisons of sociodemographic, cancer-related, health behavioral, and psychological characteristics according to DS use. With increasing age, DS use prevalence increased. Survivors with a longer time since cancer diagnosis were more likely to be a user of any DS. Survivors with a higher education level, current drinking, adequate physical activity, a comorbid disease, and a family history of cancer were more likely to use DS, and survivors with depression used DS less. Among users of any DS, subjects were more likely to be long-term users if they were younger, were of premenopausal status, received lifestyle modification counseling, had a higher cancer stage, had a shorter time since cancer diagnosis, and received more diverse cancer treatment modalities.

Table 3 presents the multivariable-adjusted associations of DS use with sociodemographic, cancer-related, health behavioral, and psychological characteristics. Education level (*p* trend = 0.002) and time lapse after cancer diagnosis (*p* trend = 0.033) were positively associated with any DS use. Alcohol use (OR [95% CI]: 1.78 [1.11–2.85]) and regular physical activity (OR [95% CI]: 1.66 [1.16–2.37]) were significantly associated with any DS use. Among DS users, survivors with a higher cancer stage (*p* trend = 0.018) and a higher number of cancer treatment modalities (OR [95% CI]: 1.66 [1.12–2.47]) were more likely to be long-term users, while survivors with a longer time since cancer diagnosis (*p* trend < 0.001) and higher FCR (OR [95% CI]: 0.45 [0.23–0.80]) showed a lower probability of taking DS long-term.

Table 4 shows multivariable-adjusted associations of any and long-term use of the four most commonly used DS with sociodemographic, cancer-related, health behavioral, and psychological characteristics. Time lapse after cancer diagnosis showed a positive association with any use of multivitamins, but it showed a consistently inverse association with long-term use of the four most frequently consumed DS (multivitamins, followed by vitamin D/calcium, vitamin C, and omega-3). The number of cancer treatment modalities had a positive association with long-term use of multivitamins and vitamin D/calcium. Low BMD was associated with both any and long-term use of vitamin D/calcium. Higher EQ-VAS was inversely associated with both any and long-term vitamin C consumption. Higher education level was positively associated with any use of multivitamins and vitamin D/calcium, but this association was not found for long-term use of DS. Family history of cancer was positively associated with any use of multivitamins and omega-3, but this association was not found for long-term use of DS. Psychological factors such as depression, anxiety, and FCR were not associated with any or long-term use of the four types of DS. 

## 4. Discussion

In this study of Korean breast cancer survivors, around half of the survivors were found to take DS for more than 2 weeks, but the rate of long-term DS use (≥6 months) was much lower (12.0%), and only one fourth of DS users were long-term users. We found that some cancer-related factors (number of cancer treatment modalities, cancer stage, time since cancer diagnosis, FCR, and EQ-VAS < 0.05) were associated with long-term use of DS, which differed from the factors associated with any DS use (education level, alcohol consumption, regular physical activity < 0.05). In addition, the factors associated with any use and long-term use of DS varied by the specific types of DS.

In spite of extensive studies on DS use among cancer survivors, the definitions of DS use in terms of its duration varied between the studies, and long-term DS use was limitedly examined. In most studies, DS use was commonly defined as taking DS for at least 2 weeks over the past 14 to 30 days or the previous year [23,24,25]. In a Korean cancer survivor study, taking any DS prior to the day of the survey was regarded as DS use [14]. In our study, we defined DS use in three ways: any use (≥2 weeks), short-term use (≥2 weeks and <6 months), and long-term use (≥6 months). To our knowledge, our study is the only study that reports the prevalence of any DS use as well as long-term DS use among Korean breast cancer survivors.

In a previous study conducted in the United States, 64.3% of 126 breast cancer patients initiated DS use after their cancer diagnosis [8]. According to a cross-sectional study based on the Advancing Survival Cancer Outcomes Trial (ASCOT), 63.6% of breast cancer survivors in the United Kingdom engaged in DS use [9]. In a single-center study of 50 American female breast cancer survivors aged 46 to 87 years old, 90% of participants were found to take one or more DS [26]. The rates of DS use observed in Western countries seem to be comparable to the rate of any DS use observed in our study. However, the rate of long-term DS use differs a lot between our study and other studies. In the Life After Cancer Epidemiology (LACE) study in the United States, 58% of early stage breast cancer patients reported DS use for at least 6 months [10]. In a French cohort mainly consisting of prostate and breast cancer survivors, 51.4% of participants were DS users, and among them, 55.6% of patients had taken DS for more than 1 year [11]. In our study, a smaller proportion of Korean breast cancer survivors was found to have used DS for 6 months or longer than was observed in Western studies. This disparity might be related not only to the characteristics of the study populations, including ethnicity or cancer stage, but also to definitions of DS use. In the LACE study, although 80% were White, Black, Hispanic and Asian participants were included, and 93% of participants were diagnosed with stage I-II breast cancer [10]. Moreover, according to the frequency of DS use, they were divided into two groups; <3 days/week and ≥3 days per week [10]. On the other hand, in a French study consisting of survivors of breast cancer (43%), prostate cancer (19%), and melanoma skin cancer (15%), the frequency of DS use was not specified [11].

DS can be defined as products intended to supplement the diet that contain one or more of the following dietary ingredients: a vitamin, a mineral, an herb or other botanical, an amino acid, or a dietary substance according to the Dietary Supplement Health and Education Act of 1994 (DSHEA 1994) [27]. In a study from the United Kingdom, calcium with or without vitamin D was the most prevalently (15.1%) consumed DS by breast cancer survivors [9]. In a study of American female breast cancer survivors, the most commonly used DS were also calcium and vitamin D to improve bone and joint health [26]. However, in a Korean study, survivors of diverse cancers, including breast cancer survivors, most frequently took multivitamins/minerals (24.6%), followed by vitamin C, omega-3 or fish oil, red ginseng, and calcium [14]. In our study, multivitamins (24.1%) were the DS most frequently consumed by Korean breast cancer survivors, followed by vitamin D/calcium (23.8%), vitamin C, and omega-3. These findings suggest that preference for DS may vary across populations, and further study is needed to investigate the reasons for the difference in preferred DS types.

In previous studies, the association of age with DS use was inconsistent. In a study of American cancer survivors, it was reported that compared to patients aged 35 to 59, patients aged 60 to 69 and 70 or older were less likely to take new DS [8]. On the other hand, older age was associated with more DS use among breast, prostate, and colorectal cancer survivors [6,9]. According to the LACE study, regular multivitamin use did not vary by age [10]. Although the association was not statistically significant in a multivariable-adjusted analysis, the direction of the relation between age and DS use in our study was opposite depending on the duration of DS use. This finding suggests that the age factor should be considered in developing strategies for adequate DS use in breast cancer survivors.

The mechanism and effect of DS use on cancer treatments itself and cancer treatment-related complications have not been clarified yet, and generally it is not recommended to take DS to prevent cancer recurrence. In our study, the number of cancer treatment modalities was positively associated with long-term DS use. It was reported that cancer survivors frequently suffer from unmet needs such as FCR, depression or anxiety, fatigue, sleep disturbance, menopausal symptoms, and weight gain, which are related to the late effects of cancer treatment [28]. Cancer patients have been known to use DS with various motives, including satisfying various unmet needs, strengthening immunity, overcoming fatigue, or improving quality of life [11,29]. Thus, we assume that the greater DS use of survivors who had received multiple modalities of cancer treatment was in an effort to overcome cancer therapy-related discomfort [30].

A previous study reported that 32.1% of DS users had a belief that DS use was important for reducing their cancer recurrence risk [9]. However, we found that breast cancer survivors with a higher level of FCR were less likely to be long-term DS users. This might be related to cancer survivors’ concern about the potential risk of DS compromising treatment effects [31]. However, it was identified that 36 types of DS, including ginseng, gingko biloba, milk thistle, vitamin A, and vitamin E, had potential adverse interactions with tamoxifen or aromatase inhibitors [32]. For vitamin D, further studies are needed to investigate its specific interactions with endocrine therapy, while its interaction with atorvastatin clearance via CYP3A4 enzyme metabolism has been reported [32,33].

Various factors were associated with any use and long-term use of each specific type of DS. For vitamin D/calcium, low BMD was positively associated with both any and long-term DS use. This finding is compatible with the finding that calcium, with or without vitamin D, is widely recommended to prevent or deter possible progression of osteopenia or osteoporosis in the course of breast cancer survivorship care [34]. We found that a longer time lapse since cancer diagnosis was inversely associated with long-term DS use, which was consistent for the four most commonly used DS. This finding may reflect a gradual decrease in the unmet needs of cancer survivors over time after treatment completion. In addition, cancer survivors may obtain more information on the adverse effects of long-term DS use, which may encourage them to withdraw from DS use.

There have been concerns about the overuse or misuse of DS by cancer survivors without medical consultation. In a study of United States cancer survivors, 46% of patients reported taking DS on their own, and only 27% asked for advice from a doctor [31]. Similarly, a French-based study also reported that 34.9% of survivors consumed DS without informing or consulting doctors [11]. Although we did not assess whether the participants had specifically discussed DS use with medical professionals, we found that lifestyle modification counseling through survivorship care was positively associated with long-term DS use, although the association was borderline significant. This finding seems to support the beneficial role of medical counseling during cancer survivorship care for raising adherence to medication.

Our study has several limitations. First, the cross-sectional study design cannot exclude the fact that some findings of the present study may reflect a reverse cause–effect relationship. Second, the study participants were recruited from university-affiliated hospitals, which means that generalization of our study findings to all Korean breast cancer survivors may be limited. Third, because we collected information on DS use using self-reported questionnaires, and 74.7% of study participants were diagnosed with breast cancer over 5 years prior to the study, there could be recall or information bias. Finally, we could not investigate the detailed reasons for DS use, and further studies including qualitative research seem necessary.

However, our study has some strengths. To our knowledge, our study is the first Asian study to evaluate DS use in terms of duration of DS use as well as specific types of DS among breast cancer survivors. In addition, a large number of breast cancer survivors participated in our research compared to previous studies, and a range of covariates were taken into consideration.

## 5. Conclusions

The prevalence of long-term DS use was only one fourth of all DS use. The factors associated with DS use differed by the duration of DS use and specific DS type. Cancer-related factors other than sociodemographic or health behavioral factors were more frequently associated with long-term DS use. These findings could be considered to develop effective strategies for the adequate DS use of breast cancer survivors.

## Figures and Tables

**Table 1:** Distribution of dietary supplement use among 693 Korean breast cancer survivors *.

Types of Dietary Supplements	Any ^(1)^ Use		Short-Term ^(2)^ Use		Long-Term ^(3)^ Use	
	Number	(%)	Number	(%)	Number	(%)
Multivitamin	167	(24.1)	139	(20.1)	28	(4.0)
Vitamin D/Calcium	165	(23.8)	123	(17.7)	42	(6.1)
Vitamin C	113	(16.3)	82	(11.8)	31	(4.5)
Omega3	87	(12.6)	63	(9.1)	24	(3.5)
Iron	10	(1.4)	7	(1.0)	3	(0.4)
CoQ10	7	(1.0)	5	(0.7)	2	(0.3)
Lactobacillus	7	(1.0)	7	(1.0)	0	(0.0)
Propolis	2	(0.3)	2	(0.3)	0	(0.0)
Ginseng	1	(0.1)	1	(0.1)	0	(0.0)
Total	334	(48.2)	251	(36.2)	83	(12.0)

* Survivors with recurrent breast cancer and secondary cancer were not included. ^(1)^ ≥2 weeks ^(2)^ 2 weeks~<6 months ^(3)^ ≥6 months.

**Table 2:** Comparisons of sociodemographic and clinical characteristics by use of dietary supplements.

Characteristics	All Participants (N = 693)			Users (N = 334)		
	Non-User	Any ^(1)^ User	*p* Value	Short-Term ^(2)^ User	Long-Term ^(3)^ User	*p* Value
Number of subjects	359 (51.8)	334 (48.2)		251 (75.1)	83 (24.9)	
Age at survey, years			0.050 ^‡^			<0.0001 ^‡^
<50	89 (59.7)	60 (40.3)		34 (56.7)	26 (43.3)	
50–54	74 (54.4)	62 (45.6)		45 (72.6)	17 (27.4)	
55–59	91 (49.2)	94 (50.8)		71 (75.5)	23 (24.5)	
60–64	42 (39.6)	64 (60.4)		53 (82.8)	11 (17.2)	
≥65	63 (53.9)	54 (46.2)		48 (88.9)	6 (11.1)	
Menopausal status			0.057			0.021
Premenopausal	70 (59.8)	47 (40.2)		29 (61.7)	18 (38.3)	
Postmenopausal	289 (50.2)	287 (49.8)		222 (77.4)	65 (22.7)	
Monthly household income, KRW			0.101 ^‡^			0.161 ^‡^
<2,000,000	67 (55.3)	54 (44.6)		42 (77.8)	12 (22.2)	
2,000,000–3,999,000	83 (50.3)	82 (49.7)		60 (73.2)	22 (26.8)	
≥4,000,000	113 (46.3)	131 (53.7)		89 (67.9)	42 (32.1)	
Unknown	96 (58.9)	67 (41.1)		60 (89.6)	7 (10.5)	
Education level			0.029 ^‡^			0.871 ^‡^
≤Middle school graduate	41 (61.2)	26 (38.8)		19 (73.1)	7 (26.9)	
High school graduate	121 (50.6)	118 (49.4)		87 (73.7)	31 (26.3)	
≥College graduate	133 (46.0)	156 (54.0)		116 (74.4)	40 (25.6)	
Unknown	64 (65.3)	34 (45.7)		29 (85.3)	5 (14.7)	
Lifestyle modification counseling			0.692			0.011
No	119 (52.9)	106 (47.1)		89 (84.0)	17 (16.0)	
Yes	240 (51.3)	228 (48.7)		162 (71.1)	66 (29.0)	
Smoking			0.729			0.337 ^†^
Never-smoker	342 (51.7)	320 (48.3)		242 (75.6)	78 (24.4)	
Ever-smoker	17 (54.8)	14 (45.2)		9 (64.3)	5 (35.7)	
Alcohol consumption			0.018			0.537
Non-drinker	320 (53.6)	277 (46.4)		210 (75.8)	67 (24.2)	
Current drinker	39 (40.6)	57 (59.4)		41 (71.9)	16 (28.1)	
Regular physical activity (≥150 min/week)			0.004			0.540
No	131 (59.8)	88 (40.2)		64 (72.7)	24 (27.3)	
Yes	228 (48.1)	246 (51.9)		187 (76.0)	59 (24.0)	
Bone mineral density			0.208			0.823
Normal (T ≥ −1.0)	105 (53.0)	93 (47.0)		73 (77.4)	21 (22.6)	
Low (T < −1.0)	167 (47.4)	185 (52.6)		141 (76.2)	44 (23.8)	
Unknown	87 (60.8)	56 (39.2)		40 (69.0)	18 (31.0)	
Body mass index, kg/m^2^			0.055 ^‡^			0.772 ^‡^
<23	165 (48.7)	174 (51.3)		131 (75.3)	43 (24.7)	
23–24.9	86 (53.8)	74 (46.3)		55 (74.3)	19 (25.7)	
≥25	96 (57.5)	71 (42.5)		55 (77.5)	16 (22.5)	
Unknown	12 (44.4)	15 (55.6)		10 (66.7)	5 (33.3)	
Comorbid disease			0.010			0.905
No	193 (56.8)	147 (43.2)		110 (74.8)	37 (25.2)	
Yes	166 (47.0)	187 (53.0)		141 (75.4)	46 (24.6)	
Living with spouse/partner			0.355			0.879
No	51 (46.8)	58 (53.2)		43 (74.1)	15 (25.9)	
Yes	283 (51.6)	265 (48.4)		199 (75.1)	66 (24.9)	
Unknown	25 (69.4)	11 (30.6)		9 (81.8)	2 (18.2)	
Family history of cancer			0.029			0.526
No	205 (55.7)	163 (44.3)		125 (76.7)	38 (23.3)	
Yes	154 (47.4)	171 (52.6)		126 (73.7)	45 (26.3)	
Cancer stage			0.553 ^‡^			0.003 ^‡^
Stage 0 *, 1	193 (52.9)	172 (47.1)		139 (80.8)	33 (19.2)	
Stage 2	125 (50.8)	121 (49.2)		88 (72.7)	33 (27.3)	
Stage 3, 4	41 (50.0)	41 (50.0)		24 (58.5)	17 (41.5)	
Time since cancer diagnosis, years			0.002 ^‡^			<0.0001 ^‡^
≤1	30 (61.2)	19 (38.8)		9 (47.4)	10 (52.6)	
2–5	78 (62.4)	47 (37.6)		22 (46.8)	25 (53.2)	
5–10	151 (50.3)	149 (49.7)		121 (81.2)	28 (18.8)	
>10	99 (45.4)	119 (54.6)		99 (83.2)	20 (16.8)	
Unknown	1 (100.0)	0 (0.0)				
Number of cancer treatment modalities ^₮^			0.761 ^‡^			0.001 ^‡^
1 (surgery)	24 (58.5)	17 (41.5)		16 (94.1)	1 (5.88)	
2	78 (48.8)	82 (51.3)		69 (84.2)	13 (15.9)	
3	166 (53.4)	145 (46.6)		106 (73.1)	39 (26.9)	
4	91 (50.3)	90 (49.7)		60 (66.7)	30 (33.3)	
Depression (K-HADS score ≥ 8)			0.021			0.280
No	177 (47.7)	194 (52.3)		150 (77.3)	44 (22.7)	
Yes	182 (56.5)	140 (43.5)		101 (72.1)	39 (27.9)	
Anxiety (K-HADS score ≥ 8)			0.442			0.604
No	284 (51.1)	272 (48.9)		206 (75.7)	66 (24.3)	
Yes	75 (54.7)	62 (45.3)		45 (72.6)	17 (27.4)	
Fear of cancer recurrence			0.576			0.652
Low (FCRI < 13)	188 (50.8)	182 (49.2)		135 (74.2)	47 (25.8)	
High (FCRI ≥ 13)	171 (52.9)	152 (47.1)		116 (76.3)	36 (23.7)	
Quality of life			0.378			0.123
Low (EQ-VAS ≤ 70)	215 (53.2)	189 (46.8)		136 (72.0)	53 (28.0)	
High (EQ-VAS: 71–100)	144 (49.8)	145 (50.2)		115 (79.3)	30 (20.7)	

Data were presented as number (%). K-HADS: Korean Hospital Anxiety and Depression Scale; FCRI: Fear of Cancer Recurrence Inventory; EQ-VAS: EuroQoL Visual Analogue Scale. (1) ≥2 weeks (2) 2 weeks~<6 months (3) ≥6 months * Stage 0: Carcinoma in situ. ₮ Total number of combined cancer treatment modalities: surgical treatment and/or other treatment (chemotherapy, radiotherapy, and hormonal therapy). *p*-value was assessed using a chi-square test or † Fisher’s exact test, after excluding participants with missing data for the relevant variables. ‡ *p*-trend was assessed using a Mantel–Haenszel chi-square test after excluding subjects with missing values for the relevant variables.

**Table 3:** Factors associated with dietary supplement use according to the duration of supplement use.

Variables	Any ^(1)^ Use (vs. No Use)		Long-Term ^(3)^ Use (vs. No or Short-Term ^(2)^ Use)		Long-Term Use (vs. Short-Term Use)	
	N = 693 (334 vs. 359)		N = 693 (83 vs. 610)		N = 334 (83 vs. 251)	
	OR (95% CI) ^†^	*p* Value	OR (95% CI) ^†^	*p* Value	OR (95% CI) ^†^	*p* Value
Age, years < 50	1.00 (ref)		1.00 (ref)		1.00 (ref)	
50–54	1.17 (0.68, 2.03)	0.566	0.89 (0.41, 1.96)	0.778	0.87 (0.33, 2.31)	0.783
55–59	1.43 (0.78, 2.60)	0.246	1.01 (0.43, 2.39)	0.980	0.86 (0.31, 2.42)	0.772
60–64	2.38 (1.20, 4.72)	0.013	0.94 (0.34, 2.56)	0.899	0.53 (0.16, 1.72)	0.290
≥65	1.21 (0.61, 2.42)	0.586	0.46 (0.14, 1.47)	0.192	0.44 (0.12, 1.65)	0.223
*p* value for trend ^‡^		0.542		0.286		0.128
Postmenopausal status	1.05 (0.62, 1.78)	0.857	0.80 (0.38, 1.67)	0.549	0.54 (0.22, 1.35)	0.189
Education level						
≤Middle school graduate	1.00 (ref)		1.00 (ref)		1.00 (ref)	
High school graduate	1.77 (0.98, 3.20)	0.058	1.40 (0.55, 3.57)	0.486	0.81 (0.26, 2.53)	0.717
≥College graduate	2.67 (1.45, 4.90)	0.002	1.47 (0.55, 3.89)	0.440	0.61 (0.19, 2.2)	0.421
*p* value for trend ^‡^		0.002		0.494		0.521
Lifestyle counseling	0.95 (0.66, 1.36)	0.781	1.61 (0.88, 2.95)	0.126	1.93 (0.96, 3.85)	0.064
Alcohol consumption	1.78 (1.11, 2.85)	0.017	1.59 (0.84, 3.02)	0.156	1.25 (0.58, 2.68)	0.573
Regular physical activity (≥150 min/week)	1.66 (1.16, 2.37)	0.006	1.28 (0.74, 2.22)	0.378	1.01 (0.52, 1.98)	0.973
Low bone mineral density (T < −1.0)	1.36 (0.92, 2.00)	0.123	1.41 (0.77, 2.59)	0.268	1.00 (0.49, 2.04)	0.998
Comorbid disease (≥1)	1.36 (0.97, 1.90)	0.071	1.26 (0.76, 2.10)	0.374	0.97 (0.51, 1.82)	0.912
Family history of cancer, yes	1.33 (0.96, 1.83)	0.082	1.34 (0.82, 2.18)	0.245	1.29 (0.72, 2.30)	0.395
Cancer stage						
Stage 0 *, 1	1.00 (ref)		1.00 (ref)		1.00 (ref)	
Stage 2	1.23 (0.86, 1.77)	0.251	1.44 (0.82, 2.54)	0.210	1.78 (0.91, 3.46)	0.092
Stage 3, 4	1.65 (0.96, 2.86)	0.073	2.44 (1.15, 5.17)	0.020	2.51 (1.01, 6,22)	0.047
*p* value for trend ^‡^		0.131		0.019		0.016
Time since cancer diagnosis, years						
≤1	1.00 (ref)		1.00 (ref)		1.00 (ref)	
2–5	0.90 (0.44, 1.83)	0.765	0.90 (0.37, 2.17)	0.813	0.87 (0.25, 3.00)	0.819
5–10	1.60 (0.82, 3.11)	0.168	0.45 (1.19, 1.07)	0.070	0.17 (0.05, 0.53)	0.003
>10	1.61 (0.80, 3.24)	0.187	0.41 (1.16, 1.05)	0.062	0.18 (0.05, 0.61)	0.006
*p* value for trend ^‡^		0.033		0.014		<0.001
Number of cancer treatment modalities ^₮^, (Increase by 1)	0.93 (0.76, 1.14)	0.491	1.32 (0.95, 1.85)	0.103	1.66 (1.12, 2.47)	0.012
Depression (K-HADS score ≥ 8)	0.74 (0.51, 1.07)	0.104	0.94 (0.54, 1.66)	0.840	1.42 (0.72, 2.77)	0.312
Anxiety (K-HADS score ≥ 8)	1.13 (0.71, 1.78)	0.610	0.72 (0.35, 1.47)	0.361	0.64 (0.27, 1.53)	0.317
Fear of cancer recurrence (FCRI score ≥ 13)	0.89 (0.62, 1.27)	0.520	0.56 (0.32, 0.97)	0.037	0.45 (0.23, 0.89)	0.021
EQ-VAS (>70)	1.00 (0.99, 1.01)	0.298	0.98 (0.97, 1.00)	0.021	0.98 (0.97, 1.00)	0.086

OR: odds ratio; CI: confidence interval; K-HADS: Korean Hospital Anxiety and Depression Scale; FCRI: Fear of Cancer Recurrence Inventory; EQ-VAS: EuroQol Visual Analogue Scale. ^(1)^ ≥2 weeks ^(2)^ 2 weeks~<6 months ^(3)^ ≥6 months * Stage 0: Carcinoma in situ. ^₮^ Number of all combined cancer treatment modality such as chemotherapy, radiotherapy, and hormonal therapy in addition to surgery. ^†^ ORs (95% CI) were estimated using multiple logistic regression analysis after adjusting for other variables listed in Table 2. ^‡^ *p*-trend was assessed by including the linear term of the relevant variables in the multiple logistic regression model.

**Table 4:** Factors associated with long-term use of four most commonly used dietary supplements.

	Multivitamin		Vitamin D or Calcium		Vitamin C		Omega-3	
	Any ^(1)^ Use vs. No Use	Long-Term ^(2)^ Use vs. Short-Term ^(3)^ Use	Any Use vs. No Use	Long-Term Use vs. Short-Term Use	Any Use vs. No Use	Long-Term Use vs. Short-Term Use	Any Use vs. No Use	Long-Term Use vs. Short-Term Use
Characteristics	OR (95% CI) ^†^	OR (95% CI) ^†^	OR (95% CI) ^†^	OR (95% CI) ^†^	OR (95% CI) ^†^	OR (95% CI) ^†^	OR (95% CI) ^†^	OR (95% CI) ^†^
Age, 1-year increase	1.03 (1.00, 1.60)	0.92 (0.83, 1.02)	1.02 (0.99, 1.05)	1.04 (0.98, 1.11)	0.99 (0.95, 1.02)	0.93 (0.84, 1.03)	0.99 (0.96, 1.03)	0.87 (0.78, 0.98)
Postmenopause	0.81 (0.45, 1.43)	1.01 (0.20, 5.09)	1.03 (0.54, 1.98)	0.28 (0.06, 1.42)	1.21 (0.62, 2.36)	0.65 (0.12, 3.54)	1.00 (0.48, 2.08)	0.61 (0.09, 4.30)
Education level increase								
≤Middle school graduate	1.00 (ref)	1.00 (ref)	1.00 (ref)	1.00 (ref)	1.00 (ref)	1.00 (ref)	1.00 (ref)	1.00 (ref)
High school graduate	2.34 (1.06, 5.18)	0.56 (0.06, 5.76)	1.60 (0.80, 3.18)	1.06 (0.24, 4.64)	1.20 (0.47, 2.57)	0.85 (0.12, 6.32)	1.18 (0.46, 3.08)	0.35 (0.03, 4.68)
≥College graduate	3.07 (1.39, 6.81)	0.51 (0.05, 5.66)	2.04 (1.01, 4.08)	0.78 (0.17, 3.55)	1.77 (0.76, 4.12)	0.87 (0.11, 6.68)	1.58 (0.61, 4.07)	0.25 (0.02, 4.04)
*p* value for trend	0.006	0.900	0.031	0.557	0.037	0.928	0.223	0.374
Lifestyle counseling	0.97 (0.64, 1.46)	2.38 (0.63, 9.05)	0.88 (0.58, 1.32)	2.28 (0.82, 6.30)	0.63 (0.40, 1.00)	1.20 (0.33, 4.33)	0.89 (0.53, 1.50)	1.31 (0.32, 5.45)
Current alcohol drinker	1.65 (1.01, 2.72)	0.72 (0.16, 3.13)	1.12 (0.65, 1.92)	3.31 (1.04, 10.5)	1.52 (0.86, 2.68)	0.85 (0.20, 3.60)	1.55 (0.84, 2.85)	1.45 (0.23, 8.94)
Regular physical activity (≥150 min/week)	1.20 (0.79, 1.82)	2.80 (0.66, 11.9)	1.32 (0.87, 2.01)	0.57 (0.22, 1.46)	1.92 (1.15, 3.21)	0.98 (0.27, 3.55)	1.33 (0.77, 2.30)	3.39 (0.68, 17.0)
Low bone mineral density (T < −1.0)	0.72 (0.46, 1.11)	1.44 (0.33, 6.27)	3.96 (2.40, 6.56)	6.70 (1.27, 38.7)	0.58 (0.36, 0.94)	1.72 (0.45, 6.59)	0.70 (0.40, 1.21)	3.87 (0.73, 20.7)
Comorbid disease (≥1)	1.15 (0.78, 1.69)	1.30 (0.41, 4.17)	1.36 (0.92, 2.00)	1.14 (0.46, 2.84)	1.01 (0.65, 1.57)	1.05 (0.30, 3.62)	1.33 (0.81, 2.18)	2.41 (0.50, 11.7)
Family history of cancer, yes	1.50 (1.04, 2.16)	1.55 (0.48, 3.05)	1.19 (0.82, 1.73)	0.84 (0.36, 1.97)	0.97 (0.63, 1.47)	0.74 (0.25, 2.23)	2.07 (1.29, 3.34)	0.96 (0.26, 3.63)
Cancer stage increase	1.17 (0.88, 1.55)	1.22 (0.49, 3.05)	1.12 (0.84, 1.49)	1.45 (0.76, 2.77)	1.11 (0.80, 1.52)	1.74 (0.79, 3.84)	0.91 (0.63, 1.32)	3.07 (0.80, 11.7)
Time since cancer diagnosis, 1-year increase	1.04 (1.00, 1.09)	0.84 (0.73, 0.97)	1.01 (0.97, 1.05)	0.86 (0.74, 0.95)	1.04 (0.99, 1.09)	0.81 (0.70, 0.94)	1.05 (0.99, 1.10)	0.85 (0.71, 1.00)
Cancer treatment modalities, increase by 1	0.83 (0.66, 1.04)	2.06 (1.05, 4.08)	1.07 (0.84, 1.36)	1.91 (1.00, 3.63)	1.14 (0.87, 1.51)	1.26 (0.60, 2.63)	0.87 (0.65, 1.16)	1.14 (0.52, 2.49)
Depression (K-HADS score ≥ 8)	0.80 (0.52, 1.21)	2.22 (0.69, 7.18)	1.19 (0.78, 1.81)	1.24 (0.45, 3.42)	0.63 (0.38, 1.03)	0.59 (0.17, 2.07)	0.70 (0.41, 1.20)	1.53 (0.35, 6.73)
Anxiety (K-HADS score ≥ 8)	1.39 (0.84, 2.31)	1.10 (0.25, 4.94)	1.03 (0.61, 1.73)	0.86 (0.28, 2.64)	1.11 (0.60, 2.05)	0.62 (0.13, 3.10)	0.96 (0.49, 1.88)	0.47 (0.06, 3.68)
Fear of cancer recurrence (FCRI score ≥ 13)	1.03 (0.69, 1.54)	0.78 (0.21, 2.85)	1.00 (0.66, 1.52)	0.42 (0.16, 1.13)	0.80 (0.50, 1.28)	0.60 (0.20, 1.83)	1.38 (0.83, 2.30)	0.48 (0.11, 2.07)
High quality of life (EQ-VAS > 70)	1.00 (0.99, 1.01)	1.00 (0.96, 1.03)	1.00 (0.99, 1.01)	0.98 (0.96, 1.01)	0.99 (0.98, 1.00)	0.97 (0.94, 0.99)	1.00 (0.99, 1.02)	0.97 (0.93, 1.01)

OR: odds ratio; CI: confidence interval; K-HADS: Korean Hospital Anxiety and Depression Scale; FCRI: Fear of Cancer Recurrence Inventory; EQ-VAS: EuroQol Visual Analogue Scale. ^(1)^ ≥2 weeks ^(2)^ 2 weeks~<6 months ^(3)^ ≥6 months ^†^ ORs (95% CI) were estimated using a multiple logistic regression analysis after adjusting for other variables listed in the table.

## Data Availability

The datasets used and analyzed in this study are available from the corresponding author on reasonable request.
